# Remarks on the BOADICEA model of genetic susceptibility to breast and ovarian cancer

**DOI:** 10.1038/sj.bjc.6602489

**Published:** 2005-03-22

**Authors:** C J van Asperen, C E Jacobi, G H de Bock

**Affiliations:** 1 Center for Human and Clinical Genetics, Department of Clinical Genetics, Leiden University Medical Center, Leiden, The Netherlands; 2 Department of Medical Decision Making, Leiden University Medical Center, Leiden, The Netherlands; 3 Department of Epidemiology, University Medical Center Groningen, Groningen, The Netherlands

**Sir**,

With much interest we read the paper by Antoniou and colleagues (*British Journal of Cancer*, October 18, p 1580). These researchers described the development of a genetic model for familial breast cancer, named BOADICEA, which takes into account the simultaneous effects of BRCA1, BRCA2 and other genes. There is a considerable need for such predictions for purposes of management of women referred to genetic clinics and for the considerations of eligibility of any prophylactic interventions in both the clinical and research settings.

The BOADICEA model requires a computer program for risk estimation, which is not easy to use in clinical practice. Moreover, such a model is mostly hard to understand for clinicians. Understandable risk estimation should, however, be available to clinicians, so that they know what they are actually doing while estimating disease risks. Our concern about this aspect of the BOADICEA model was illustrated in Figure 3. The authors presented a woman aged 40 years and unaffected with cancer. Her mother and maternal aunt developed ovarian cancer and two other maternal aunts developed breast cancer before the age of 50 years. This family is a good example of a typical Hereditary Breast and Ovarian Cancer (HBOC) family. The risk for breast cancer in these families is increased because of the combination of both breast and ovarian cancers in the family, which highly points to BRCA1 mutations. The BRCA1 mutation probability as predicted by the BOADICEA model is high, 40.9%. The model then predicted her risk of breast cancer at only 13%. For a clinician, this risk estimation is hard to understand and feels illogical. It seems that in the risk prediction by Antoniou *et al* (2004) the presence of ovarian cancer is not important for the estimation of the breast cancer risk for this woman. The authors did not discuss this point.

Recently, Jonker *et al* (2003) have published a genetic model to predict someone's breast cancer risk based on the family history of breast and ovarian cancer. This model can be considered as an extension of the Claus model combined with the BRCAPRO model (Claus *et al*, 1991; Parmigiani *et al*, 1998). In the Jonker *et al* model, the familial clustering of breast and ovarian cancer is explained by three genes, *BRCA1*, *BRCA2* and a hypothetical third gene *BRCAu*. This third gene was modelled to explain all familial clustering of breast cancer unaccounted for by the *BRCA1* and *BRCA2* genes. The model parameters were estimated using published estimates of population incidence and relative risks.

As well as the BOADICEA model, the Jonker *et al* model is not easy to use in clinical practice. For this reason, we extended the easy-to-use Claus tables into the ‘Claus plus method’ based on the Jonker model (Claus *et al*, 1994; Van Asperen *et al*, 2004). Our method uses the Claus tables, but also incorporates information on the presence of ovarian cancer, bilateral breast cancer and whether there are more than two affected relatives. The formula we obtained simplifies risk estimation in familial breast cancer: 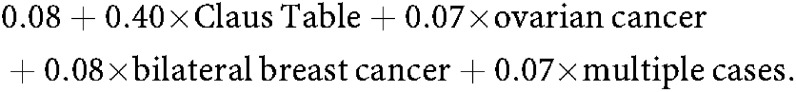 The formula starts with an intercept of 0.08. This is the population risk for breast cancer and the basis for further risk estimation. The value of the Claus table should be multiplied by 0.4. The formula subsequently includes the information on ovarian cancer, bilateral breast cancer and more than two affected relatives. These characteristics in the formula are one or zero. This new method might offer a good alternative for breast cancer risk estimation in clinical practice. The ‘Claus plus method’ is an easy applicable method for hand-written pedigrees and at the moment it is widely used in the Dutch cancer clinics. Based on the ‘Claus plus method’, the predicted risk of breast cancer of the 40-year-old woman in Figure 3 in the Antoniou paper was 31%. Although we are still working on the validation of our method, this risk figure is more in agreement with the risks as observed in typical HBOC families.

The authors have mentioned several improvements to be made for their multi-purpose BOADICEA model like genotype-specific incidence rates, risk for other cancers and allele frequencies. Besides this, we would like to recommend paying attention to the applicability for clinical practice. To attain full development for the BOADICEA model, the authors should be in close contact with the ultimate users in clinical practice.
